# Repetitive tooth-clenching triggers kynurenine pathway activation in masseter muscle of women with temporomandibular disorders myalgia

**DOI:** 10.1097/PR9.0000000000001466

**Published:** 2026-08-14

**Authors:** Golnaz Barjandi, Jacob Ahlberg Weidenfors, Lilly Schwieler, Sophie Erhardt, Sofia Louca Jounger, Malin Ernberg

**Affiliations:** Departments of a Dental Medicine and; b Physiology and Pharmacology, Karolinska Institutet, Sweden

**Keywords:** Kynurenine, Tryptophan, Quinolinic acid, 3-Hydroxykynurenine, Nicotinamide, Temporomandibular disorders, Myalgia, Microdialysis, Skeletal muscle, Exercise

## Abstract

**Background:**

Inflammation alters the kynurenine pathway (KP), producing metabolites related to pain regulation. Previous microdialysis studies indicate increased levels of inflammatory markers in temporomandibular disorders myalgia (TMDM) that increase in response to tooth-clenching. This study aimed to elucidate the role of the KP in TMDM by investigating intramuscular KP metabolites in response to tooth-clenching.

**Methods:**

This study included 41 women; 15 TMDM myofascial pain with referral (MFP), 13 TMDM local myalgia (MYA), and 13 controls (CTR). Microdialysis was applied to the masseter muscle for 220 minutes to analyze tryptophan (TRP), l-kynurenine (KYN), 3-hydroxykynurenine (3-HK), quinolinic acid (QUIN) and nicotinamide (NAM). After baseline (140 minutes), participants performed a 20-minute tooth-clenching exercise followed by 1 hour of recovery. Pain variables were assessed throughout the experiment.

**Results:**

NAM levels increased significantly in response to tooth-clenching across all groups (*P* < 0.001), while TRP, KYN, QUIN, and 3-HK increased only in MFP (*P* < 0.001 to *P* = 0.041). Supervised multivariate analysis showed that changes in TRP, KYN, and QUIN levels in response to exercise, and NAM levels at baseline and recovery, significantly separated MFP from CTR and MYA (*P* = 0.040). In addition, decreased concentration levels of mainly NAM and TRP were significantly associated with increased widespread pain (*P* = 0.02) and pain during recovery (*P* = 0.030).

**Conclusion:**

These findings indicate that intramuscular KP metabolites increase in response to tooth-clenching, particularly in MFP, distinguishing them from both CTR and MYA. This supports the link between intramuscular inflammation and exercise-induced alterations in the KP, providing further insights in the TMDM pathophysiology and differentiation of its subdiagnosis.

## Abstract 2

Supplemental Digital Content is Available in the Text.

Patients with regional, but not local, temporomandibular disorders myalgia show elevated intramuscular kynurenine pathway activation in response to exhaustive tooth-clenching, suggesting differential peripheral mechanisms.

## 1. Introduction

Temporomandibular disorders (TMD), particularly TMD myalgia (TMDM), are common chronic orofacial pain conditions affecting 3% to 15% of the population, with a female predominance.^10,46,54^ TMDM is characterized by masticatory muscle pain and limited jaw function,^41^ but often extends beyond physical symptoms, causing emotional distress, functional impairment, and reduced life quality.^29,34,59^ The Diagnostic Criteria for TMD (DC/TMD) categorizes TMDM into local myalgia (MYA) and myofascial pain with referral (MFP) based solely on whether pain on palpation remains local (MYA) or spreads beyond the palpated muscle (MFP, regional pain).^41^ Recent findings show that MFP and MYA also differ in pain and psychosocial characteristics, likely affecting their large variations in treatment response.^1,55,60^ Understanding the peripheral mechanisms across TMDM subdiagnosis may reduce chronicity and improve prognosis.

The pathogenesis of TMDM is complex, with subgroup differences largely unexplored. Chronic stress and muscle overloading, with subsequent tissue trauma, are proposed as contributing factors.^49,51^ These may increase the release of inflammatory and endogenous pain-producing substances, leading to activation and sensitization of peripheral and central pain receptors, including N-methyl-d-aspartate (NMDA) receptors. If prolonged and combined with impaired pain inhibition, such alterations may contribute to pain chronification.^31,52^

To investigate these mechanisms under controlled conditions, repetitive tooth-clenching have been used to transiently induce or increase jaw muscle pain, fatigue, and intramuscular biochemical changes in pain-free individuals and patients with TMDM.^17,27^ Similar to peg-board exercise in trapezius myalgia,^13^ it increases the release of inflammatory markers in the masseter,^28^ supporting its use in understanding TMDM mechanisms. Moreover, it enables standardized investigation of exercise-induced intramuscular biochemical changes.

Chronic stress and inflammation can also upregulate the kynurenine pathway (KP), through elevated indoleamine-2,3-dioxygenase (IDO) activity, shifting tryptophan (TRP) metabolism toward the KP.^32^ This may increase neurotoxicity through NMDA agonist quinolinic acid (QUIN) and/or reduce neuroprotection, via NMDA antagonist kynurenic acid (KYNA), potentially contributing to pain mechanisms.^32,37^ Systemic KP alterations have been associated with TMDM comorbidities,^16^ including fibromyalgia, irritable bowel syndrome, migraine, and depression.^4,14,57^ Similarly, our pilot data have linked lower plasma TRP levels and higher IDO activity to increased TMDM pain intensity.^2^

To date, the intramuscular KP has not been examined in chronic myalgia.^8^ In healthy skeletal muscle, long-term aerobic exercise may activate the neuroprotective KP, contributing to the benefits of exercise.^30^ Conversely, acute intense exercise may activate the neurotoxic KP,^20^ possibly due to inflammatory cytokines and hypoxia.^21,36,40^ Examining intramuscular KP metabolites in TMDM may therefore provide insights into mechanisms underlying its development and subdiagnoses.

This study aimed to explore KP metabolites in the masseter muscle of MFP, MYA, and pain-free controls (CTR) during repetitive tooth-clenching^12,27^ and in correlation with pain and psychosocial variables. The null hypotheses (H_0_) were that KP metabolites do not differ between groups, change during tooth-clenching or correlate with psychosocial or pain-related variables.

## 2. Materials and methods

### 2.1. Procedure

**Figure 1.**
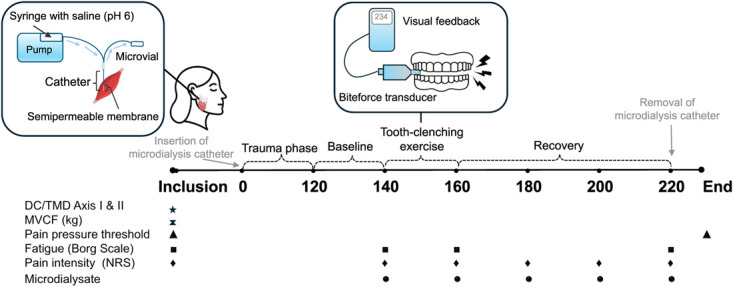
Flowchart of the experimental procedure for all included participants with timepoints (minutes) of the examination according to the diagnostic criteria for TMD (DC/TMD) axes I and II, assessment of maximal voluntary clenching force (MVCF, kg), pain pressure threshold, fatigue (borg scale), pain intensity (NRS), and collection of microdialysates. The microdialysis was conducted over 220 minutes structured in 4 phases; trauma phase (0–120 minutes), baseline (140 minutes), tooth-clenching (160 minutes), and recovery (180–220 minutes). The collection of microdialysates at baseline, tooth-clenching, and recovery was selected for analysis.

On inclusion, participants were examined using the validated DC/TMD Axis I (clinical examination) and II (questionnaire)^41^ along with a brief medical history, maximal voluntary clenching force (MVCF), and pressure pain thresholds (PPT). Intramuscular microdialysis was performed for 220 minutes with samples collected every 20 minutes. After 140 minutes (baseline), a 20-minute tooth-clenching exercise was performed. Pain intensity and fatigue was assessed throughout the microdialysis (Fig. 1).

### 2.2. Participants

Participants were recruited from Orofacial Pain Clinics at the University Dental Clinic Karolinska Institutet (Huddinge, Sweden) or through advertisement.

Patients with TMDM (n = 28) were included with the inclusion criteria: MYA (n = 13) or MFP (n = 15) diagnosis according to the DC/TMD,^41^ 18 years and older, and pain duration ≥3 months.

Thirteen age-matched CTR were included with the inclusion criteria: good general health, 18 years and older, and no orofacial pain.

Given the female predominance in TMDM,^10,46^ men were excluded to reduce sex-related confounders. Additional exclusion criteria comprised systemic inflammatory disease, whiplash-associated disorders, neuropathic pain, cranial neuralgias, dental pain, other severe somatic or psychiatric conditions, pregnancy, excessive alcohol consumption, and using analgesics or hypnotics 48 hours before the experiment. Exclusions were determined based on patient history and DC/TMD assessments, performed by a calibrated dentist.

#### 2.2.1. Ethical approval

This study was conducted in accordance with the Declaration of Helsinki and Good Clinical Practice and approved by the Regional Ethics Review Board in Stockholm (2018/1614-31/2, 2021-06893-02, 2021-06966-01). On inclusion, all participants received information about the study and signed an informed consent.

### 2.3. Assessment

Pain and psychosocial variables were assessed with validated and widely used patient-reported outcome measures presented below.

#### 2.3.1. Pain-related variables

The Graded Chronic Pain Scale (GCPS) v.2 (1-month version) assessed the current, average, and worst orofacial pain intensity over the past month on a numerical rating scale (NRS) 0 to 10 (0 = no pain and 10 = worst pain imaginable).^41,43,56^ The mean of these scores, multiplied with 10, yielded the Characteristic Pain Intensity (CPI, range 0-100).

The Widespread Pain Index (WPI) assessed pain in 19 body regions over the past 7 days.^5,62^ The total pain regions (range 0-19) were calculated as WPI with scores above 5 indicating a widespread pain.^61^

Pain intensity was assessed every 20 minutes during the microdialysis using an NRS 0 to 10, with the same end points as for the GCPS.

Fatigue was assessed at 0-, 140-, 160-, and 220 minutes during the microdialysis using the Borg Rating of Perceived Exertion Scale (range 6-20).

Pressure pain thresholds (PPT) was recorded before and after the microdialysis using an electronic algometer (Somedic Sales AB, Höör, Sweden). It was applied with a standardized pressure of 50 kPa/second in the most painful region of the masseter muscle and on the ipsilateral index finger for extracranial reference. In cases of bilateral pain or no pain, PPT was applied on the dominant side. Participants were instructed to press a button when the pressure became painful. Three trials were performed per site and the mean value was used for analysis.

#### 2.3.2. Psychosocial variables

The Patient Health Questionnaire-9 (PHQ-9, range 0-27) assessed depressive symptoms over the past 2 weeks, with cutoffs of 5, 10, 15, and 20 indicating mild, moderate, moderately severe, and severe depression levels, respectively.^22^

The Generalized Anxiety Disorder scale (GAD-7, range 0-21) assessed anxiety symptoms over the past 2 weeks,^48^ with cutoffs of 5, 10, and 15 indicating mild, moderate, and severe anxiety levels, respectively.^48^

The Patient Health Questionnaire-15 (PHQ-15, range 0-30) assessed somatic symptoms over the past month, with cutoffs at 5, 10, and 15 indicating mild, moderate, and severe somatic symptom, respectively.^9,23^

The Pain Catastrophizing Scale (PCS, range 0-52) assessed thoughts and feelings during pain,^50^ with cutoffs of 20 and 30 indicating risk and high risk of pain catastrophizing, respectively.^50^

The Perceived Stress Scale (PSS-10, range 0-40) assessed stress levels over the past month with cutoffs of 13 and 21 indicating moderate and high stress, respectively.^35^

The Insomnia Severity Index (ISI, range 0-28) assessed sleep disturbances over the past 2 weeks, with cutoffs of 8, 15, and 22 indicating mild, moderate, and severe insomnia, respectively.^3^

#### 2.3.3. Maximal voluntary clenching force

Maximal voluntary clenching force (MVCF) (kg) was measured using a bite-force transducer (Aalborg University, Denmark) placed between the first molar on the microdialysis side. Participants were instructed to clench their teeth with maximum effort for 2 to 3 seconds across 3 trials. The mean of these trials was calculated and used as MVCF during the tooth-clenching exercise.

### 2.4. Microdialysis procedure

Microdialysis is an established in vivo technique for sampling interstitial substances with minimal tissue trauma. It is based on passive diffusion of molecules across a semipermeable membrane, enabling continuous assessment of local biochemical changes during steady-state conditions.^44^

Before initiating the procedure, topical anesthesia (0.5 mL Lidocaine, Xylocaine 20 mg/mL) was applied to the skin overlying the masseter muscle for 20 to 30 minutes. A sterile, splitable introducer was then inserted into the most prominent point of the muscle (identified through palpation) at a 45° angle to an initial∼10 mm depth. On reaching resistance at muscle fascia, the introducer was advanced an additional 30 mm. A sterile 100-kDa microdialysis catheter (71 high cut-off brain microdialysis catheter, M Dialysis AB, Johanneshov, Sweden) with a 30-mm semipermeable membrane was inserted to its full depth. The introducer was then removed by splitting its plastic sheath. The catheter was perfused with an isotonic perfusion fluid containing NaCl, KCl, and CaCl (pH ∼6, P000034 Perfusion Fluid T1, M Dialysis AB, Johanneshov, Sweden) at 5 µL/min using a microinfusion pump (CMA 107).

The participants rested between 0 and 120 minutes to allow tissue recovery (ie, trauma phase), and then an additional 20-minute rest (140 minutes, set as baseline). Between 140 and 160 minutes, a standardized tooth-clenching exercise was performed, alternating 30 seconds of clenching at 50% MVCF (guided by visual feedback) with 30 seconds of rest for 20 minutes. This exercise was used as it has shown to induce low pain levels in healthy individuals, increase pain in TMDM, and inflammatory markers in both healthy individuals and patients with TMDM.^17,27^ After the exercise, the participants rested for 1 hour (180-220 minutes, ie, recovery), after which the catheter was removed. Dialysate samples were collected every 20 minutes and stored at −80°C until biochemical analysis (Fig. 1).

Specific timepoints were selected for analysis to explore potential variations in KP metabolites during the different physiological phases; baseline (140 minutes, representing steady-state interstitial metabolite concentrations), during tooth-clenching exercise (Δ_160–140_ and Δ_180–140_, reflecting the acute impacts during and directly after exercise), and recovery (mean value of 180–220 minutes, reflecting the prolonged recovery).

### 2.5. Recovery test

Three independent in vitro recovery tests were performed using a standard solution of 10 KP metabolites (nicotinamide (NAM), picolinic acid (PIC), QUIN, KYNA, TRP, l-kynurenine (KYN), and 3-hydroxykynurenine (3-HK)). Initial concentrations were set at 10 µM for all metabolites except for TRP (at 10 nM). To resemble physiological concentrations, 50 µL of the solution was mixed with 450 µL of physiological NaCl. A sterile microdialysis catheter (71 high cut-off brain microdialysis catheter) was immersed in the solution and perfused using a microinfusion pump (CMA 107) with a flow rate of 5 µL/min. Samples were collected every 20 minutes for 160 minutes and stored at −80°C.

### 2.6. Ultra-performance liquid chromatography—tandem mass spectrometry

Microdialysate sample (n = 496) data for analytes TRP, KYN, QUIN, KYNA, 3-HK, PIC, and NAM were acquired by a targeted ultra-performance liquid chromatography—tandem mass spectrometry (UPLC-MS/MS) method developed for cerebrospinal fluid. Sample data were acquired in 6 separate assays run over 7 days (2023-03-15 to 2023-03-22). For full description of the UPLC-MS/MS method including sample preparation and stability tests for all metabolites, see Refs. 42 and 53. In brief, samples were analyzed using a Xevo TQ-XS triple-quadrupole mass spectrometer (Waters, Manchester, United Kingdom) equipped with a Z-spray electrospray interface and a Waters Acquity UPLC I-Class FTN system (Waters, MA). MS analysis was performed using electrospray ionization in positive ion mode, with data acquired using multiple reaction monitoring (MRM). MS parameters were set as follows: source temperature 150°C, capillary voltage +3.0 kV, desolvation temperature 650°C, desolvation gas flow 1000 L/hour. The detector gain was set to 1. The UPLC system was equipped with an Acquity HSS T3 2.1 × 150 mm, 1.8 μm column (Waters, Product Number [PN]: 186,003,540) set to a temperature of 50°C. The method used 2 mobile phases composed of A: 0.6% formic acid in water and B: 0.6% formic acid in methanol (UPLC grade). An isolator column (Waters, 2.1 × 50 mm column, PN: 186,004,976) was installed to retain contaminants from the mobile phase. The flow rate was set at 0.3 mL/minute and the run time for each sample was 13.0 minutes. The m/z for the MRM transitions of each individual analyte were: TRP, 206 > 118; KYN, 209 > 94; KYNA, 190 > 116; QUIN, 168 > 78; 3-HK, 225 > 110; PIC, 124 > 78; NAM, 123 > 78; and for the internal standards (IS): TRP-d3, 208 > 118.8; KYN-d4, 213 > 94; QUIN-d3, 171 > 81; KYNA-d5, 195 > 121; 3-HK-d3, 228 > 163; PIC-d4, 128 > 82; NAM-[13C6], 129 > 101.

For most microdialysate samples, metabolites were detected at concentrations higher than the lowest level of quantification (LoQ, CSF: TRP, 5 nM; KYN, 0.5 nM; KYNA, 0.5 nM; QUIN, 1 nM; 3-HK, 0.5 nM; PIC, 0.5 nM; NAM, 0.5 nM). In exception to this, 4 measurements of 3-HK, 10 measurements of QUIN, 10 measurements of PIC, and 356 measurements KYNA detected levels below the respective analyte LoQ. Coefficients of variance (%CV) for quality control samples within assays were less than 10% (exception:<15% for PIC in 2 of 6 assays) for all metabolites measured. %CV for quality control samples between different assays were less than 10% (exceptions:<15% for PIC, NAM) for all metabolites measured.

### 2.7. Statistical methods

Standard statistical analyses were performed using SigmaPlot v.14.0 (Systat Software, Inc., San Jose, CA) and GraphPad Prism 8. A 2-tailed *P*-value <0.05 was considered statistically significant.

Data normality was evaluated using the Shapiro–Wilk test and visual inspection of Q-Q plots. KP metabolites were not normally distributed and were log-transformed before analysis.

ANOVA or Kruskal–Wallis test (if ordinal data) with Bonferroni–Dunn correction was used for group comparisons of background characteristics.

To account for the repeated-measure design, linear mixed-effects models with restricted maximum likelihood (REML) and Geisser–Greenhouse correction were used to evaluate changes in KP metabolites and pain variables over time. Time, group, and their interaction were included as fixed effects and subjects as a random intercept. Model assumptions were evaluated by visual inspection of residuals (Q-Q plots). Missing data were handled within the mixed-effect modelling. Least square means, mean differences, and 95% confidence intervals (CI) were derived from the models. Multiple comparisons were adjusted by controlling the False Discovery Rate or Bonferroni correction.

To explore interrelationships among KP metabolites across the different phases of microdialysis, supervised multivariate data analyses were performed using SIMCA-P+ (version 18.0; Umetrics Inc., Umeå, Sweden), following previous recommendations.^58^ All variables were mean-centred, scaled, and, if needed, log-transformed. Orthogonal partial least squares regression (OPLS/OPLS-DA) was used to analyse group membership, psychosocial, and pain variables using metabolites during baseline, tooth-clenching exercise (Δ_160–140_ and Δ_180–140_), and recovery as regressors (X-variables).

The OPLS-DA was chosen over multiple linear regression, as it handles the interdependency and multicollinearity among highly correlated variables (such as KP metabolites) without compromising model stability and validity.

Model validity was assessed by permutation testing (999 permutations) and cross-validation analysis of variance (CV-ANOVA). CV-ANOVA was further used to calculate a *P*-value for each model, also determining the statistical significance of the model (*P* < 0.05).^58^

## 3. Results

### 3.1. Recovery test

All metabolites showed large losses (∼10%) as concentrations decreased over time in 2 trials (20 minutes: 14.8%, 160 minutes: 1.64%), while one trial showed more stable results (40 minutes: 9.94%, 160 minutes: 11.95%). Owing to larger losses of PIC and KYNA, these were not measured in the participant dialysate samples.

### 3.2. Background data

Results are summarized in Table 1. No significant group differences were found for age or BMI (*P* = 0.7). Compared with CTR, MFP had higher PHQ-9, GAD-7, PHQ-15, PCS, and PSS scores (*P* < 0.05 to <0.001) whereas MYA only reported higher scoring for PHQ-15 (*P* < 0.05). MYA and MFP reported no significant differences regarding psychosocial variables.

**Table 1:** Descriptive characteristics of the study participants.

	MFP n = 15	MYA n = 13	CTR n = 13
Age (yr)	28.5 (±5.4)	26.7 (±5.1)	28.1 (±8.4)
Pain duration (yr)	7.0 (5.5)*,†	2.0 (4.3)*	0.0 (0)
BMI (kg/m^2^)	22.4 (8.6)	23.0 (4.5)	23.0 (4.7)
NRS (0–10)	5.0 (5.0)*	3.0 (0.0)*	0.0 (0)
CPI (0–100)	60.0 (33.0)*,†	40.0 (11.5)*	0 (0)
MVCF 50% (kg)	83.0 (20.0)*,†	135.0 (20.5)	148.0 (36.0)
WPI (0–19)	7.0 (5.0)*,†	4.0 (4.0)†	1.0 (2.5)
PHQ-9 (0–27)	8.0 (10.0)*	2.0 (7.5)	2.0 (5.0)
GAD-7 (0–21)	9.0 (7.0)*	3.0 (6.5)	1.0 (2.5)
PHQ-15 (0–30)	14.0 (10.0)*	10.0 (9.5)*	3.0 (7.0)
PCS (0–56)	9.0 (23.0)*	7.0 (12.0)	3.0 (6.5)
PSS (0–40)	19.0 (8.0)*	15.0 (9.5)	11.0 (14.0)
ISI (0–28)	14.0 (14.0)	5.0 (7.5)	9.0 (10.5)

Mean values (± standard deviation) or median values (interquartile ranges) are shown. ANOVA/ANOVA on ranks were used for the analysis of group differences. Statistical significance (adjusted for multiple comparison with Bonferroni–Dunn correction) is indicated with a capital letter if *P* < 0.001 or with a lower case letter if *P* < 0.05.

* Significant compared with CTR.

† Significant compared with MYA.

MFP = myofascial pain with referral; MYA = local myalgia; CTR = pain-free controls; BMI = body mass index; NRS = numerical rating scale; CPI = characteristic pain intensity; MVCF = maximal voluntary clenching force; WPI = Widespread Pain Index; PHQ = Patient Health Questionnaire; GAD-7 = Generalized Anxiety Disorder 7-item scale; PCS = Pain Catastrophizing Scale; PSS-10 = Perceived Stress Scale, 10 items; ISI = Insomnia Severity Index.

MFP reported increased pain duration, CPI, and WPI compared with both CTR (*P* < 0.001) and MYA (*P* < 0.05). CPI and pain duration was increased in MYA versus CTR (*P* < 0.001).

### 3.3. The effect of tooth-clenching on pain variables

#### 3.3.1. Pain intensity and fatigue

**Figure 2.**
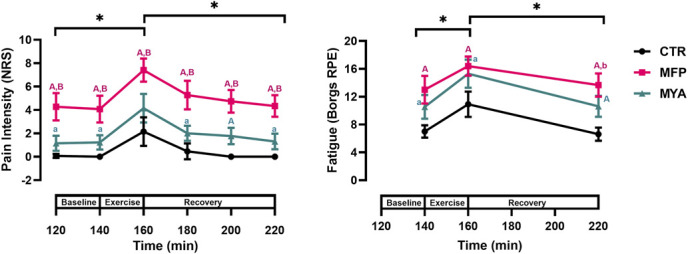
(A) Pain intensity (NRS, 0–10) and (B) fatigue (Borg Rating of Perceived Exertion scale, 6–20) at baseline (140 minutes), during tooth-clenching (160 minutes), and recovery (180–220 minutes) in 41 women (13 with local myalgia [MYA], 15 myofascial pain with referral [MFP], and 13 pain-free controls [MYA]). Data are presented as estimated marginal means with 95% confidence intervals derived from linear mixed-effects. Multiple comparisons were adjusted using the Bonferroni correction test. Adjusted significant differences are indicated with uppercase letters if *P* < 0.001 or with lowercase letters if *P* < 0.05, where A/a = significant vs CTR and B/b = significant vs MYA.

Results are presented in Figures 2A and B, with full pairwise comparisons in Supplemental digital content (see Table 1, http://links.lww.com/PR9/A423). Pain intensity and fatigue increased during tooth-clenching and decreased during recovery in all groups (MFP *P* < 0.002, MYA *P* < 0.002, CTR *P* < 0.003).

MFP reported higher pain intensity than CTR and MYA across all time points (*P* < 0.001). Similarly, MYA showed increased pain intensity at baseline and recovery (*P* < 0.003) with a non-significant increase during tooth-clenching (*P* = 0.58).

MFP and MYA reported higher fatigue compared with CTR at all time points (MFP *P* < 0.001, MYA *P* ≤ 0.005). MFP showed higher fatigue compared with MFP during recovery (*P* < 0.022), but not at baseline or tooth-clenching (*P* = 0.160 to *P* > 0.999).

#### 3.3.2. Pressure pain thresholds

Detailed results are summarized in Table 2. At the masseter site, PPT decreased during the experiment in all groups (*P* < 0.001). Compared with CTR, MFP had decreased PPT (masseter) throughout the experiment (*P* < 0.001), whereas an MYA showed decreased PPT values only at start (*P* = 0.016).

**Table 2:** Pressure pain thresholds derived from linear mixed-effects models.

Comparison	LS mean diff	95% CI	Adj. *P*
Masseter			
Start			
MFP vs CTR	−100.4	−147.5 to −53.2	**<0.001**
MYA vs CTR	−57.1	−105.9 to −8.3	**0.016**
MYA vs MFP	43.3	−3.9–90.4	0.083
End			
MFP vs CTR	−79.8	−127.0 to −32.7	**<0.001**
MYA vs CTR	−46.9	−95.7–1.9	0.064
MYA vs MFP	32.9	−14.3–80.1	0.275
Start vs end			
CTR (within-group)	75.3	45.7–104.8	**<0.001**
MFP (within-group)	54.7	27.2–82.2	**<0.001**
MYA (within-group)	65.1	35.5–94.6	**<0.001**
Reference			
Start			
MFP vs CTR	−132.9	−245.4 to −20.4	**0.015**
MYA vs CTR	−99.0	−215.5–17.4	0.122
MYA vs MFP	33.9	−78.6–146.4	>0.999
End			
MFP vs CTR	−101.3	−213.8–11.2	0.091
MYA vs CTR	−79.3	−195.7–37.2	0.299
MYA vs MFP	22.1	−90.5–134.6	>0.999
Start vs end			
CTR (within-group)	63.5	26.8–100.2	**0.001**
MFP (within-group)	31.9	−2.3–66.1	0.067
MYA (within-group)	43.7	7.0–80.4	**0.021**

Data are presented as least squares (LS) mean differences with 95% confidence intervals and adjusted *P*-values (Bonferroni correction) derived from linear mixed-effects models including time and group as fixed effects and subject as a random intercept. Negative values indicate lower PPT in the first group compared with the reference group. Significant results are highlighted in bold.

At the reference site, PPT decreased during the experiment in both CTR (*P* = 0.001) and MYA (*P* = 0.021), but not in MFP (*P* = 0.067). MFP showed lower PPT at the reference site compared with CTR at start (*P* = 0.02). No significant group differences in PPT were observed at reference site after the experiment.

Regardless of site or timepoint, no significant differences between MFP and MYA were observed (*P* = 0.083 to >0.999).

### 3.4. The effect of tooth-clenching exercise on kynurenine metabolites

#### 3.4.1. Standard statistics

**Figure 3.**
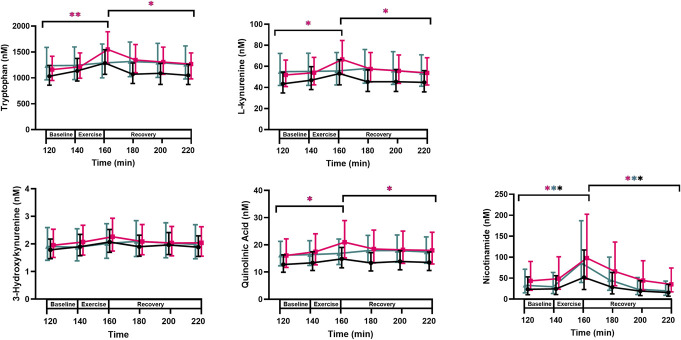
Interstitial concentrations of tryptophan, l-kynurenine, 3-hydroxykynurenine, quinolinic acid, and nicotinamide during microdialysis at baseline (140 minutes), during a 20-min tooth-clenching exercise (160 minutes), and during recovery (180–220 minutes) in 41 women (13 with local myalgia, MYA, 15 with myofascial pain with referral MFP, and 13 pain-free controls, CTR). Data are presented as back-transformed estimated marginal means with 95% confidence intervals derived from linear mixed-effects models FDR correction. Adjusted significant differences are indicated with uppercase letters if *P* < 0.001 or with lowercase letters if *P* < 0.05, where A/a = significant vs CTR and B/b = significant vs MYA. Data points are marginally moved to assist interpretation.

Results are presented in Figure 3. All groups showed increased NAM levels during tooth-clenching (160 vs 140 minutes; CTR: least square mean difference [MD] 2.31, *P* = 0.01; MFP: MD 2.01, *P* = 0.001; MYA: MD 2.93, *P* < 0.001) with corresponding decreases during recovery (220 vs 160 minutes; CTR: MD 0.30, *P* = 0.001; MFP: MD 0.36, *P* = 0.001; MYA: MD 0.23, *P* < 0.001).

Only MFP had significantly increased TRP (MD 1.27, *P* = 0.002), KYN (MD 1.23, *P* = 0.004), and QUIN (MD 1.20, *P* = 0.02) during tooth-clenching with corresponding decreases during recovery (220 vs 160 minutes; TRP: MD 0.83, *P* = 0.005; KYN: MD 0.82, *P* = 0.004; QUIN: MD 0.87, *P* = 0.02).

No significant group differences were found during baseline, tooth-clenching, or recovery.

#### 3.4.2. Multivariate regression to determine group membership using kynurenine metabolites as regressors

Detailed results are summarized in Table 3. Significant separation of MFP and CTR subgroups were observed, driven by changes in TRP, KYN, and QUIN levels postexercise (Δ_180-140_) and NAM levels during baseline and recovery. Similarly, changes in TRP, KYN, 3-HK, and QUIN metabolite levels during exercise (Δ_160-140_) and NAM levels at baseline and recovery were found to be important in driving the significant separation of MFP from MYA. No significant model was found for MYA vs CTR.

**Table 3:** Orthogonal partial least squares-DA regressions of group memberships using the tryptophan and kynurenine metabolite levels as regressors.

CTR vs MYA			CTR vs MFP			MFP vs MYA		
Substance	VIP	*P* (corr)	Substance	VIP	*P* (corr)	Substance	VIP	*P* (corr)
NAM Δ_160-140_	2.49	0.91	NAM _recovery_	2.17	0.56	NAM _baseline_	1.97	0.66
NAM _recovery_	2.16	0.91	NAM _baseline_	2.14	0.58	NAM _recovery_	1.84	0.59
NAM _baseline_	1.95	0.83	NAM Δ_160-140_	1.60	0.34	KYN Δ_160-140_	1.49	0.76
NAM Δ_180-140_	1.06	0.59	TRP Δ_180-140_	1.33	0.74	NAM Δ_160-140_	1.41	0.39
			QUIN Δ_180-140_	1.20	0.63	TRP Δ_160-140_	1.36	0.80
			KYN Δ_180-140_	1.11	0.61	3 HK Δ_160-140_	1.27	0.70
						QUIN Δ_160-140_	1.26	0.65
R^2^X = 0.424			R^2^X = 0			R^2^X = 0.229		
R^2^Y = 0.157			R^2^Y = 0.418			R^2^Y = 0.224		
Q^2^Y = 0.153			Q^2^Y = 0.375			Q^2^Y = 0.22		
*P* (CV-ANOVA) = 0.190			*P* (CV-ANOVA) = 0.040			*P* (CV-ANOVA) = 0.040		

OPLS-DA regressions of group memberships (CTR vs MYA, CTR vs MFP and MFP vs MYA) using the tryptophan and kynurenine metabolite levels at baseline (at 140 min), in response to exercise (Δ_160-140_ and Δ_180-140_) and at recovery (180-220 min) as regressors (X-variables). For each regression model, R^2^Y, Q^2^, VIP, *P* (corr), and the *P*-value from CV-ANOVA are reported in the bottom rows. R^2^Y; total variation in Y explained by the model, Q^2^; the model's predictive accuracy. VIP; the importance of variables in driving group separation. *P* (corr); correlation coefficient. VIP >1 and *P* (corr) > 0.4 to 0.5 indicates significant metabolites. In CTR vs MYA and CTR vs MFP, a positive *P* (corr) denotes higher values for MYA or MFS compared with CTR, and in MFS vs MYA, it indicates higher values for MFS. The CV-ANOVA *P*-value indicate model validity and the significance of group separation, *P* < 0.05 is considered significant.

OPLS-DA = Orthogonal partial least squares regression-discriminant analysis; CTR = Pain-free participants; MYA = local myalgia; MFP = myofascial pain with referral; NAM = nicotinamide; TRP = tryptophan; QUIN = quinolinic acid; KYN = kynurenine; 3-HK = 3-hydroxykynurenie.

The OPLS-DA models showed a modest but significant fit (CV-ANOVA, Table 3). Permutation testing (999 permutation) further confirmed model validity and predictive performance, supporting the observed group separation (supplemental digital content, Fig. 1, http://links.lww.com/PR9/A423).

#### 3.4.3. Multivariate regression of pain and psychosocial variables using kynurenine pathway metabolites as regressors

Detailed results are summarized in Table 4. Clinical pain intensity during recovery was negatively associated with TRP and NAM at recovery, baseline, and NAM levels during exercise. Hence, high pain intensities in patients during the prolonged recovery from tooth-clenching were associated with low levels of TRP and NAM at these timepoints and small changes in NAM during exercise.

**Table 4:** Orthogonal partial least squares regressions of pain intensity and widespread pain index using tryptophan and kynurenine metabolite concentrations as regressors.

NRS recovery period		Widespread pain index	
Substance	VIP (*P* (corr))	Substance	VIP (*P* (corr))
NAM Δ_160-140_	2.32 (−0.78)	NAM Δ_160-140_	2.31 (−0.62)
NAM _recovery_	1.83 (−0.72)	NAM _recovery_	1.55 (−0.48)
NAM _baseline_	1.69 (−0.68)	3-HK Δ_180-140_	1.32 (−0.77)
TRP _recovery_	1.02 (−0.75)	TRP Δ_180-140_	1.31 (−0.65)
KYN _baseline_	1.02 (−0.72)	QUIN Δ_180-140_	1.24 (−0.65)
TRP _baseline_	1.01 (−0.73)	NAM _baseline_	1.15 (−0.37)
		QUIN _recovery_	1.04 (−0.52)
R^2^X = 0.34		R^2^X = 0.22	
R^2^Y = 0.22		R^2^Y = 0.34	
Q^2^Y = 0.15		Q^2^Y = 0.25	
*P* (CV ANOVA) = 0.030		P (CV-ANOVA) = 0.020	

OPLS regressions, in pain patients (ie, MYA + MFP), of pain intensity and WPI using metabolite concentrations at baseline (140 min), in response to exercise (Δ_160-140_ and Δ_180-140_), and at recovery (180-220 min) as regressors (X-variables). For each regression model, R^2^Y, Q^2^, VIP, *P* (corr), and the *P*-value from CV-ANOVA are reported in the bottom rows. R^2^Y; the total variation in Y explained by the model, Q^2^; the model's predictive accuracy. VIP indicate the importance of variables in driving group separation. *P* (corr); correlation coefficient. VIP >1 and *P* (corr) > 0.4 to 0.5 indicates significant metabolites. The CV-ANOVA *P*-value indicate model validity and the significance of group separation, *P* < 0.05 is considered significant. A positive coefficient indicates that this variable will be associated with high values and vice versa.

OPLS-DA = Orthogonal partial least squares regression-discriminant analysis; MYA = local myalgia; MFP = myofascial pain with referral; NAM = nicotinamide; TRP = tryptophan; QUIN = quinolinic acid; KYN = kynurenine; 3-HK = 3-hydroxykynurenie.

WPI showed negative association with KP metabolites in the pain group. The most important regressor here was NAM (Δ_180-140_ and at recovery). 3-HK, TRP, and QUIN postexercise (Δ_180-140_) were also negatively associated with WPI (Table 4). No other significant associations were identified between KP metabolites and clinical pain or psychosocial severity measures.

## 4. Discussion

This is the first study to examine intramuscular KP metabolites in chronic myalgia. We found significant increases in TRP, KYN, and QUIN during tooth-clenching followed by decreases in recovery, only in MFP patients, distinguishing them from MYA and CTR. Conversely, NAM increased during exercise and decreased during recovery across all groups, suggesting a common metabolic response to muscle activity independent of diagnosis. Using this standardized tooth-clenching model, previously shown to increase pain and inflammation in TMDM,^27^ our findings provide novel insights into the peripheral KP in chronic myalgia, suggesting a link between intramuscular KP and inflammation.

While previous studies have not explored KP metabolites in painful skeletal muscle,^8^ our findings align with systemic KP alterations observed in other pain populations.^16^ Decreased KYNA to QUIN and 3-HK ratios have been reported in fibromyalgia and chronic fatigue syndrome,^14^ indicating activity of the neurotoxic KP branch.^14^ Similarly, elevated QUIN levels observed in our MFP cohort suggests localized neurotoxic KP activation, likely driven by proinflammatory cytokines.^16,49^ This increase may worsen pain through excitotoxicity, oxidative stress, and inflammation,^37^ while reducing neuroprotective KYNA levels and its analgesic effects.^32,37^ However, these mechanisms remain speculative in TMDM, as KYNA was not measured in our study. Nevertheless, serum KP metabolites have previously been linked to inflammation and muscle dysfunction.^18^ Together with previous microdialysis studies, showing increased intramuscular proinflammatory cytokines, serotonin, and glutamate (NMDA receptor agonist) in TMDM,^12,27^ our findings support a potential association between neurotoxic KP activation, muscle inflammation, and NMDA activity in MFP pain. Our supervised multivariate analysis further showed that clenching-induced changes in neurotoxic KP metabolites distinguished MFP from CTR and MYA, suggesting possible differences in their inflammatory profiles. Enhanced inflammation in MFP may promote neurotoxic KP activation and pain sensitization,^49^ raising the hypothesis that MYA and MFP may involve different pain mechanisms despite both being TMDM conditions. Tooth-clenching as an experimental model may induce acute muscle responses relevant to TMDM but does not reflect its full complexity, warranting further research to validate these findings and clarify underlying mechanisms.

Moreover, MFP is characterized by increased pain distribution, pain sensitivity, and potentially stronger influence of central sensitization.^25,31,55,60,64^ The significantly lower PPT in masseter and reference site in MFP compared with CTR supports involvement of central sensitization. By contrast, MYA did not differ from CTR at the reference site, indicating potential peripheral sensitization. Together with the more severe pain profile in MFP and intermediate symptoms in MYA, this suggests that MFP may represent a more advanced TMDM stage, further supporting a severity gradient across subgroups in this cohort.^1,55,60^

The significant changes in NAM levels observed in all groups throughout the experiment, suggest a general response to muscle activity rather than a condition-specific effect. NAM is a precursor to nicotinamide adenine dinucleotide (NAD+), an important cofactor in cellular-, energy-, and repair processes,^38,65^ especially in metabolically active tissues (eg, skeletal muscles). NAM is recycled to NAD + through a salvage pathway mediated by nicotinamide phosphoribosyltransferase (NAMPT),^39,65^ and both are essential in maintaining NAD + levels.^38,65^ Intramuscular NAMPT deficiency have shown muscle degeneration and weakness, while its overexpression preserves NAD + levels, muscle mass, and function.^11^ Impaired NAMPT activity may also result in NAM accumulation through inefficient recycling.^6^ Although NAM levels did not differ significantly between our study groups, the highest concentrations were observed in MFP, possibly reflecting greater muscle degeneration and reduced muscle strength (lower MVCF). This is supported by preliminary data from our group, where bulk RNA sequencing of masseter biopsies shows mild inflammation and muscle degeneration in MYA, but more profound muscle degeneration in MFP (Akopian et al., personal communication). While the role of NAM and NAD+ in chronic myalgia remains unexplored,^8^ the exercise-induced increases in our cohort may reflect an adaptive response to oxidative stress and inflammation,^27,51^ potentially preserving NAD + levels and supporting tissue recovery. Nicotinamide has previously also been associated with reduced proinflammatory cytokines, oxidative stress,^38,47,65^ and protection against skeletal muscle atrophy,^15^ suggesting a potential role in masticatory muscle physiology.

The interstitial NAM and TRP levels correlated negatively with pain intensity during recovery and WPI. Despite conflicting findings in the literature, this aligns with our pilot data linking low TRP levels with increased TMD pain intensity,^2,8^ supporting the potential influence of TRP on pain mechanisms. NAM may also have a protective role in pain modulation, as it is proposed to reduce pain and swelling in arthritis inflammation^19,33^ and protect against diabetic neuropathy.^26^ Moreover, the prolonged pain during the recovery may reflect hyperalgesia, potentially suggesting a role for these metabolites.

### 4.1. Study limitations

This study has several limitations that need to be addressed. The small, only female sample limits generalizability, increasing the risk of type 1 and II errors.^45^ Accordingly, supervised multivariate findings should be considered exploratory and hypothesis-generating. As mentioned, the tooth-clenching task in our study was used to possibly induce acute changes in metabolite levels, similar to the peg board used in microdialysis studies of trapezius myalgia.^12,13^ Thus, it was not used as an experimental model to evoke TMDM pain, as in some other studies.^7,24^ Moreover, potential confounders, including comorbidities, physical activity, and lifestyle factors may have influenced our findings, particularly given the small homogeneous sample. Finally, methodological limitations prevented assessment of certain KP metabolites, e.g. KYNA and PIC, which could have provided better understanding of the KP in TMDM. Future studies should consider perfusion fluids including albumin to improve metabolite recovery and detection sensitivity during microdialysis.^63^

### 4.2. Conclusion

This study showed that tooth-clenching induces dynamic changes in intramuscular KP metabolites, particularly in MFP, providing insights into the KP in TMDM, its subgroup differentiation, and the potential role of NAM in masticatory muscle physiology. However, larger, sex-balanced studies are warranted to validate our findings.

## Disclosures

The authors have no conflict of interest to declare.

## Supplemental digital content

Supplemental digital content associated with this article can be found online at http://links.lww.com/PR9/A423.
