# Supplementary material for: Repetitive tooth-clenching triggers kynurenine pathway activation in masseter muscle of women with temporomandibular disorders myalgia
Source: Pain Rep. 2026 Aug 14;11(5):e1466. doi: 10.1097/PR9.0000000000001466 (PMC13480753; doi:10.1097/PR9.0000000000001466)
Supplement: Supplementary file 1 [file painreports-11-e1466-s001.pdf]

Supplementary Table 1. Pairwise comparisons of pressure pain thresholds (PPT) derived from linear mixed-effects model

	Comparison	LS mean diff.	95% CI	Adj. p-value
**Pain intensity**				
140				
	MFP vs. CTR	4.07	2.60 to 5.53	**<0.001**
	MYA vs. CTR	1.23	0.45 to 2.01	**0.002**
	MYA vs. MFP	-2.84	-4.42 to -1.26	**<0.001**
160				
	MFP vs. CTR	5.25	3.36 to 7.13	**<0.001**
	MYA vs. CTR	2.00	-0.058 to 4.05	0.058
	MYA vs. MFP	-3.25	5.13 to 1.36	**<0.001**
220				
	MFP vs. CTR	4.33	3.16 to 5.51	**<0.001**
	MYA vs. CTR	1.31	0.45 to 2.16	**0.003**
	MYA vs. MFP	-3.03	-4.39 to -1.67	**<0.001**
160 vs. 140				
	CTR (within-group)	2.15	0.09 to 4.21	**0.037**
	MFP (within-group)	3.33	1.93 to 4.74	**<0.001**
	MYA (within-group)	2.92	0.61 to 5.24	**0.009**
220 vs. 160				
	CTR (within-group)	-2.15	-4.21 to 0.09	**0.037**
	MFP (within-group)	-3.08	-4.42 to 1.71	**<0.001**
	MYA (within-group)	-2.85	-5.10 to -9.59	**0.009**
**Fatigue**				
140				
	MFP vs. CTR	6.00	3.33 to 8.68	**<0.001**
	MYA vs. CTR	3.54	1.21 to 5.86	**0.002**
	MYA vs. MFP	-2.46	-5.57 to 0.65	0.160
160				
	MFP vs. CTR	5.48	2.76 to 8.19	**<0.001**
	MYA vs. CTR	4.39	1.16 to 7.60	**0.005**
	MYA vs. MFP	-1.09	-4.02 to 1.83	>0.999
220				
	MFP vs. CTR	7.05	4.72 to 9.37	**<0.001**
	MYA vs. CTR	4.00	1.87 to 6.13	**<0.001**
	MYA vs. MFP	-3.05	-5.7 to -0.37	**0.022**
160 vs 140				
	CTR (within-group)	3.92	1.40 to 5.40	**<0.003**
	MFP (within-group)	3.40	1.47 to 5.33	**0.001**
	MYA (within-group)	4.77	1.83 to 7.71	**0.002**
220 vs 160				
	CTR (within-group)	-4.31	-6.79 to -1.82	**0.001**
	MFP (within-group)	-2.73	-4.40 to -1.07	**0.002**
	MYA (within-group)	-4.69	-7.01 to -2.37	**<0.001**

Data are presented as mean differences with 95% confidence intervals (CI) and adjusted p-values (Bonferroni correction for multiple comparisons). Significant results are highlighted in bold.

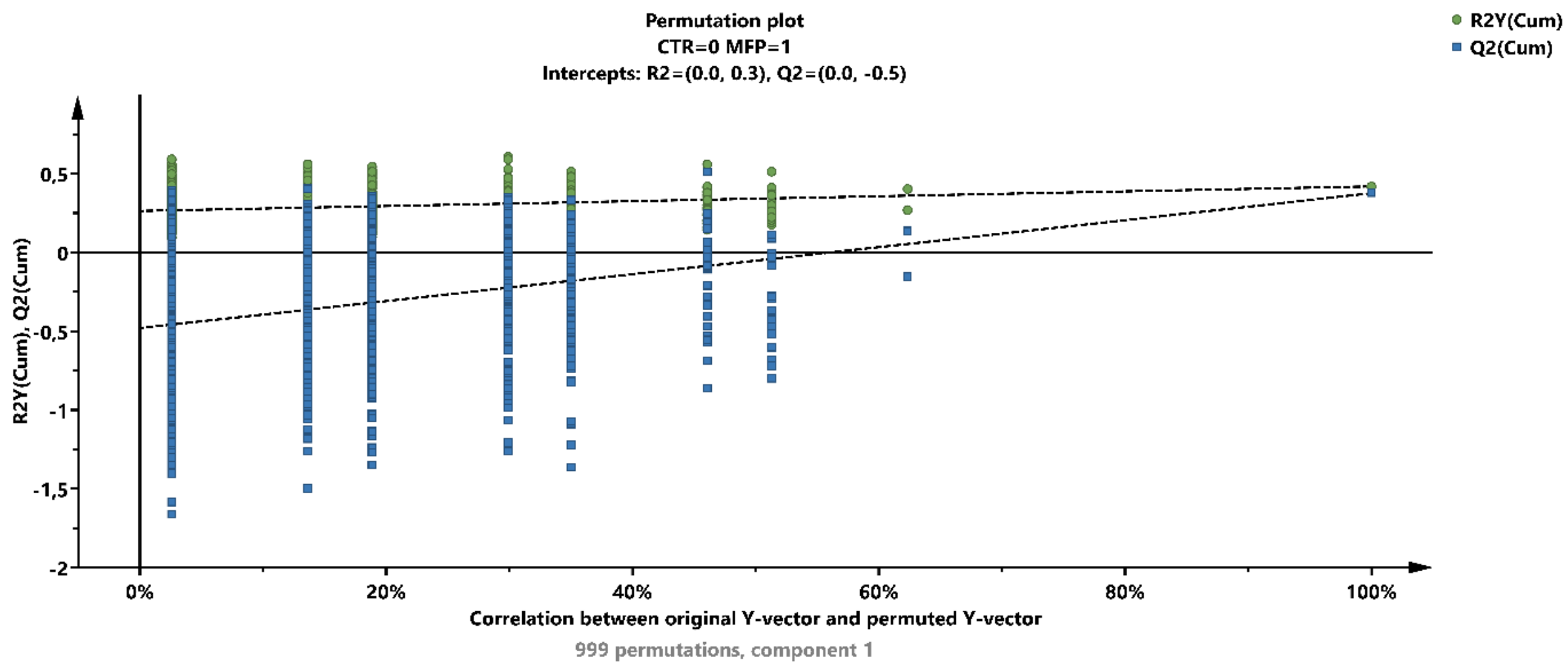

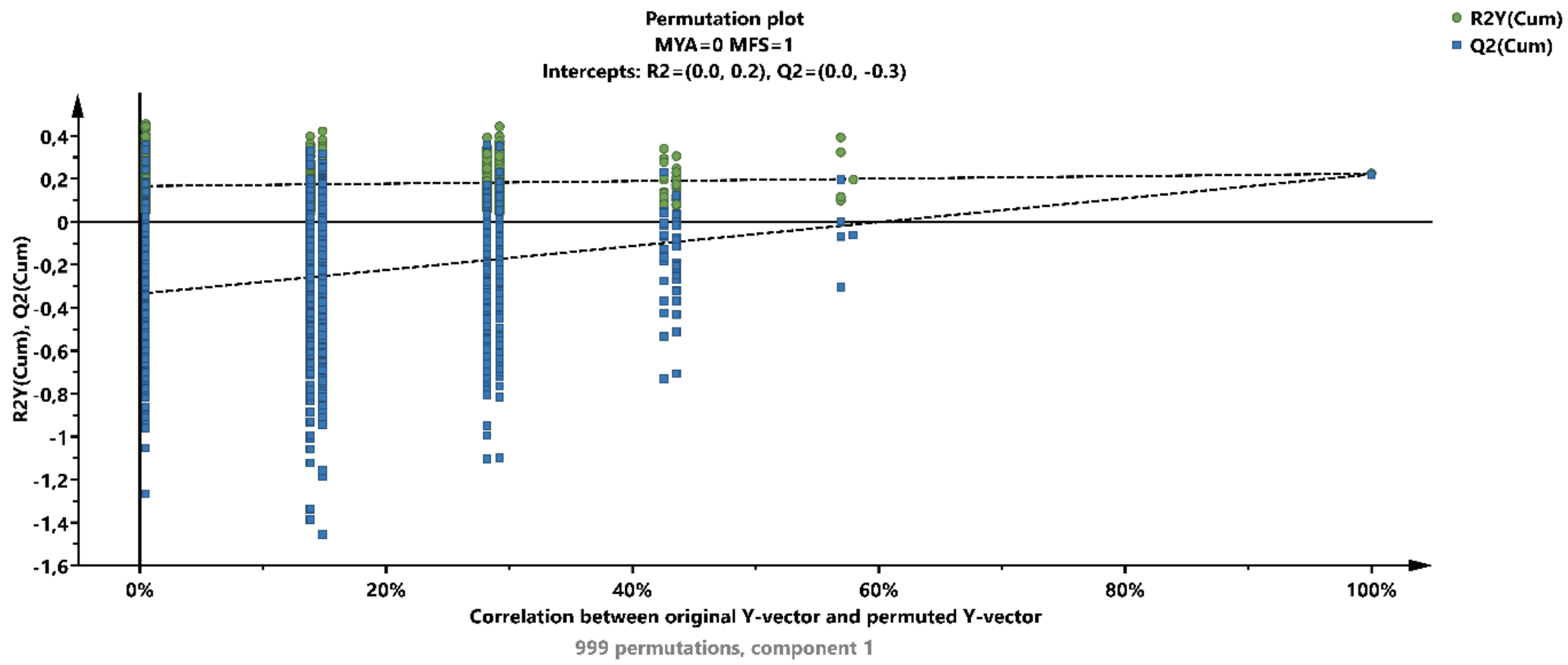

Suppl. Fig. 1. Permutation plots (999 permutations) to assess model validity. The negative Q^2<0 confirms the validity of OPLS models and indicate that the model is not overfitted. although R^2 is modest.
